# Pancreatitis-associated mortality in the United States: A population-based analysis of trends and disparities, 1999 to 2023

**DOI:** 10.1097/MD.0000000000048969

**Published:** 2026-05-22

**Authors:** Rateeba Qadri, Muzamil Khan, Hasiba Karimi, Usman Itoo, Hasibullah Aminpoor, Dhruvan Patel

**Affiliations:** a Department of Internal Medicine, Mercy Catholic Medical Center, Darby, PA; b Faculty of Medicine, Bezmialem Vakif University, Istanbul, Turkey; c Faculty of Medicine, Kabul University of Medical Sciences “Abu Ali Ibn Sina,” Kabul, Afghanistan; d Department of Internal Medicine, Rabia Balkhi National Complex Hospital, Kabul, Afghanistan.

**Keywords:** alcohol-related pancreatitis, CDC WONDER database, epidemiologic trends, health disparities, pancreatitis-associated mortality

## Abstract

Pancreatitis contributes substantially to gastrointestinal mortality, yet its population-level burden remains incompletely characterized. We conducted a retrospective, population-based study using the Centers for Disease Control and Prevention Wide-Ranging Online Data for Epidemiologic Research Multiple Cause-of-Death database (1999–2023). Adults aged ≥45 years with pancreatitis (International Classification of Diseases, Tenth Revision: K85.x, K86.0–K86.1) listed anywhere on the death certificate were included. Age-adjusted mortality rates were calculated using the 2000 US standard population. Temporal trends were analyzed using Joinpoint regression to estimate annual percent change (APC) and average APC. The age-adjusted mortality rate declined from 6.30 to 4.63 per 100,000, with an overall decrease (average APC: −1.18%). Mortality declined through 2018, increased during 2018 to 2021, and declined thereafter. Higher mortality was observed among males, older adults, Black and American Indian/Alaska Native populations, and in the Southern United States. Alcoholic pancreatitis mortality increased, while chronic pancreatitis mortality remained stable. Pancreatitis-associated mortality declined over 2 decades but showed a temporary reversal from 2018 to 2021. Persistent disparities and rising alcohol-related mortality highlight the need for targeted prevention and equitable care strategies.

## 1. Introduction

Pancreatitis remains a major contributor to gastrointestinal morbidity and mortality in the United States. As an inflammatory disorder of the pancreas, it can range from a self-limited illness to a severe systemic disease complicated by organ failure, infection, and death. Acute pancreatitis is among the most common causes of gastrointestinal hospitalization nationwide, while chronic pancreatitis is characterized by recurrent admissions, progressive pancreatic insufficiency, and a sustained risk of adverse outcomes over time.^[1–3]^ Beyond its acute clinical impact, pancreatitis is associated with long-term complications, including diabetes, malnutrition, and functional decline, all of which contribute to diminished quality of life and increased vulnerability to mortality.^[4,5]^ Despite substantial advances in supportive care, imaging, critical care management, and standardized treatment protocols, pancreatitis continues to impose a significant burden on the US healthcare system. It is consistently ranked among the leading gastrointestinal causes of hospitalization and readmission, reflecting both its high incidence and its potential for recurrent or severe disease.^[6]^ Improvements in early recognition and management have translated into better short-term outcomes for many patients; however, these gains may not be evenly distributed across populations, etiologic subtypes, or care settings.^[7]^

Many national mortality studies focus on specific pancreatitis subtypes, an approach that may incompletely capture the actual mortality burden due to pancreatitis. Given the frequent clinical and coding overlap between acute and chronic disease, as well as variability in etiologic attribution over time, analyzing pancreatitis as a unified condition provides a more stable and comprehensive assessment of pancreatitis-associated mortality and improves the reliability of long-term trend and subgroup analyses.^[8]^ Moreover, relatively few studies have examined pancreatitis mortality using multiple cause-of-death data, which more accurately reflects the contribution of pancreatitis to fatal outcomes beyond cases in which it is listed as the underlying cause of death.^[9]^ Consequently, updated evaluations are needed to define the true population-level mortality burden due to pancreatitis and to determine whether long-standing improvements in outcomes have been sustained in recent years or altered by evolving healthcare delivery, population risk factors, and pandemic-era disruptions.

Accordingly, we conducted a comprehensive, population-based analysis of overall pancreatitis-associated mortality in the United States from 1999 through 2023 using national death certificate data. The objectives of this study were to characterize long-term trends in age-adjusted mortality and to examine disparities by sex, age, race and ethnicity, geographic region, place of death, disease type, and etiology, with particular attention to recent deviations from historical patterns. By extending mortality surveillance through 2023 and evaluating pancreatitis as a unified condition, this study provides an updated and robust assessment of the evolving epidemiology of pancreatitis-related mortality in the United States.

## 2. Materials and methods

### 2.1. Study setting and population

We conducted a retrospective, population-based analysis of pancreatitis-associated mortality in the United States using data from the Centers for Disease Control and Prevention Wide-Ranging Online Data for Epidemiologic Research (CDC WONDER) Multiple Cause-of-Death database. This publicly available database contains deidentified national death certificate records and captures all conditions listed on the death certificate, enabling assessment of diseases that contribute to death beyond the designated underlying cause. The study follows the Strengthening the Reporting of Observational Studies in Epidemiology guidelines.

Deaths occurring between January 1, 1999, and December 31, 2023, were included if pancreatitis was listed as a multiple cause of death (any mention). Pancreatitis-related deaths were identified using International Classification of Diseases, Tenth Revision (ICD-10) codes for acute pancreatitis (K85.x) and chronic pancreatitis (K86.0–K86.1). Analyses were restricted to individuals aged ≥45 years to reduce instability from sparse death counts at younger ages and to minimize the impact of CDC WONDER data suppression on age-adjusted mortality estimates. Accordingly, findings reflect pancreatitis-associated mortality among middle-aged and older adults.

Variables extracted included year of death, age, sex, race and ethnicity, US Census region, and place of death. In accordance with CDC WONDER confidentiality policies, mortality counts fewer than 10 within a given stratum are suppressed; strata affected by suppression were excluded from stratified trend analyses to ensure statistical reliability.

### 2.2. Study design and variable definitions

Mortality data were examined overall and stratified by calendar year, sex, age group (45–54, 55–64, 65–74, 75–84, and ≥85 years), race and ethnicity, US Census region, place of death, disease type, and etiology. Race and ethnicity were categorized as White, Black or African American, Hispanic or Latino, Asian or Pacific Islander, American Indian or Alaska Native, and more than 1 race, as defined within CDC WONDER. Geographic analyses were conducted using the 4 US Census regions (Northeast, Midwest, South, and West). Place of death was classified as inpatient medical facility, outpatient or emergency department, dead on arrival, home, nursing home or long-term care facility, hospice facility, and other or unknown locations. Disease type was classified as acute or chronic pancreatitis based on ICD-10 coding, with categories defined to be mutually exclusive. Etiology-specific analyses categorized deaths as alcohol-induced pancreatitis when alcohol-specific ICD-10 codes (K85.2 or K86.0) were present. All remaining pancreatitis-associated deaths were classified as nonalcoholic pancreatitis, representing a heterogeneous group that includes biliary, metabolic, medication-related, procedural, and idiopathic etiologies. In addition to subtype-specific analyses, pancreatitis was examined as a unified condition to provide a comprehensive assessment of pancreatitis-associated mortality, reduce misclassification related to subtype attribution, and improve the stability and interpretability of long-term trend and disparity analyses.

Race- and ethnicity-specific analyses were conducted separately for 2 periods (1999–2017 and 2018–2023). This stratification was implemented to account for changes in race and ethnicity reporting practices in national mortality data, to improve the classification of multiracial identities in more recent years, and to reduce instability in long-term trend estimation in the post-2018 and pandemic-era periods. Separating analyses into these intervals improved the interpretability and statistical reliability of race- and ethnicity-specific mortality patterns while minimizing bias related to reporting changes and sparse cell counts.

### 2.3. Statistical analysis

Age-adjusted mortality rates (AAMRs) were calculated using the direct standardization method with the 2000 US standard population as the reference. Temporal trends in pancreatitis-associated mortality were evaluated using Joinpoint Regression Program (National Cancer Institute, Bethesda) with log-linear modeling to estimate annual percent change (APC) for individual time segments and average APC (AAPC) over the full study period, each reported with 95% confidence intervals (CIs). Models allowed for up to 4 joinpoints, consistent with standard practice for long-term national trend analyses. Statistical significance was defined as 95% CIs that did not include 0. Trend analyses were conducted overall and stratified by sex, age group, race and ethnicity, US Census region, place of death, disease type (acute vs chronic pancreatitis), and etiology (alcoholic vs nonalcoholic pancreatitis). No individual-level clinical, socioeconomic, or treatment-related covariates were available in the CDC WONDER database.

To assess whether subgroup-specific trends reflected artifacts of age adjustment or true epidemiologic changes, we conducted additional age-stratified analyses separately for alcoholic and nonalcoholic pancreatitis. Trends were evaluated within predefined age groups (45–54, 55–64, 65–74, 75–84, and ≥85 years) using crude mortality rates and Joinpoint regression. We also examined absolute death counts over time to determine whether rate-based trends were supported by corresponding changes in mortality burden. Nonalcoholic pancreatitis was defined as pancreatitis-related deaths without alcohol-specific ICD-10 codes and therefore represents a residual, heterogeneous category. Age-specific estimates based on unreliable crude rates or suppressed counts were excluded. Accordingly, estimates of alcoholic pancreatitis among adults aged ≥75 years were not reported due to instability of rates.

### 2.4. Data sharing and ethics

This study utilized publicly available, deidentified data from the CDC WONDER database. Institutional review board approval and informed consent were not required, as the study did not involve human subjects as defined by federal regulations.

## 3. Results

### 3.1. Overall pancreatitis-associated mortality trends

**Figure 1.**
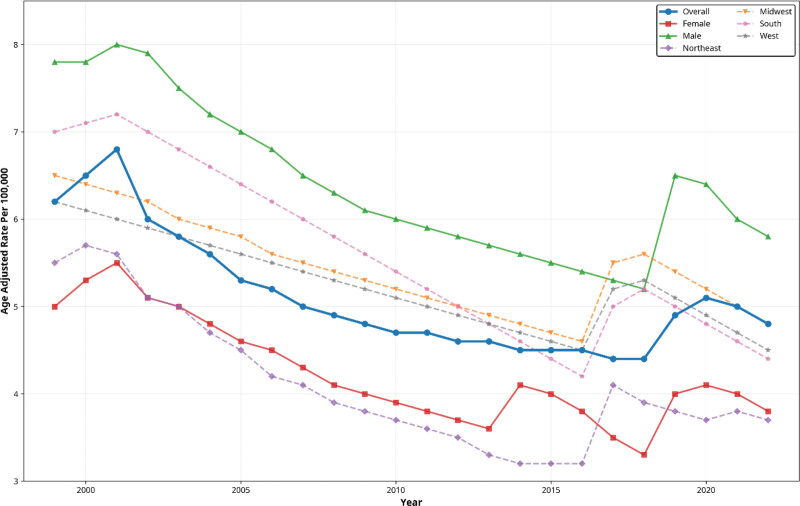
Age-adjusted mortality rates per 100,000 population from 1999 to 2023, stratified by sex (female and male) and census region (Northeast, Midwest, South, and West), showing overall declining trends with regional and sex-specific variations.

**Figure 2.**
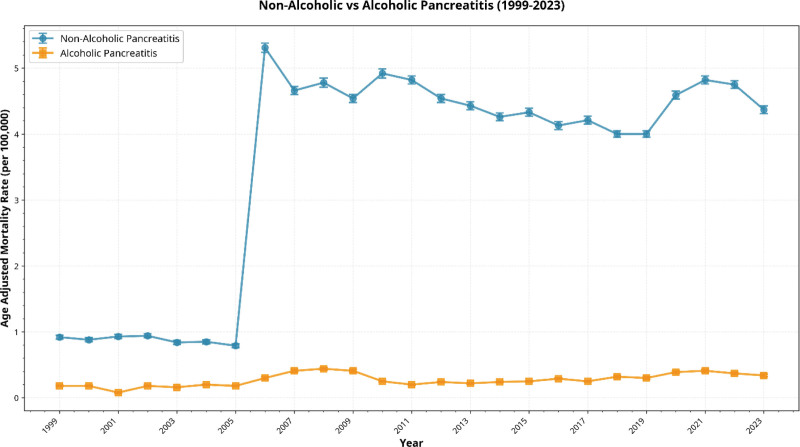
Age-adjusted mortality rates per 100,000 population from 1999 to 2023, stratified by alcoholic versus nonalcoholic pancreatitis.

**Figure 3.**
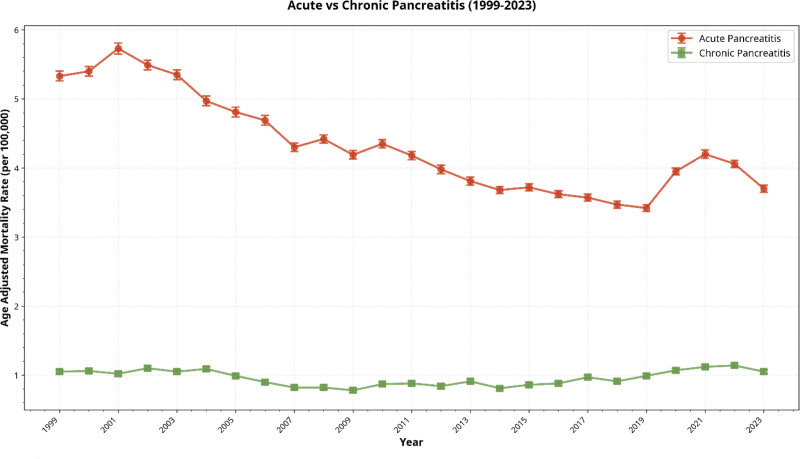
Age-adjusted mortality rates for acute and chronic pancreatitis in the United States from 1999 to 2023 demonstrate that acute pancreatitis carries a substantially higher mortality burden, with a mean rate of 4.34 per 100,000 compared with 0.78 to 1.14 per 100,000 for chronic pancreatitis.

Between 1999 and 2023, a total of 156,339 pancreatitis-associated deaths were recorded in the United States. The AAMR declined from 6.30 in 1999 to 4.63 in 2023, with a mean AAMR of 5.22 over the study period. Overall mortality declined significantly across the full interval (AAPC: −1.18%, 95% CI: −1.46 to −0.91; Table 1, Figs. 1–3).

**Table 1:** Age-adjusted mortality rates (AAMRs) and temporal trends in pancreatitis-associated mortality in the United States from 1999 to 2023, stratified by census region, age group, sex, etiology (alcoholic vs nonalcoholic), and pancreatitis subtype (acute vs chronic).

Variable	AAMR 1999		AAMR 2023	Overall AAMR		Time period	APC (95% CI)	AAPC (95% CI)
Overall	6.30		4.63	5.22		1999–2001	3.85* (0.06–7.67)	−1.18* (−1.46 to −0.91)
						2001–2007	−4.04* (−6.19 to −3.22)	
						2007–2018	−1.92* (−2.43 to −1.08)	
						2018–2021	7.23* (4.69–8.75)	
						2021–2023	−5.36* (−8.60 to −2.17)	
Census region								
Northeast	5.55		3.77	4.23		1999–2018	−3.08* (−3.58 to 2.66)	−1.75* (−2.15 to −1.37)
						2018–2021	9.14 (−3.55 to 11.68)	
						2021–2023	−4.47 (−9.60 to 3.95)	
Midwest	6.25		5.06	5.36		1999–2018	−2.02* (−2.51 to −1.59)	−1.09* (−1.49 to −0.72)
						2018–2021	7.98 (−2.75 to 10.28)	
						2021–2023	−5.09 (−10.29 to 2.92)	
South	7.02		4.92	5.72		1999–2015	−2.90* (−4.30 to −2.22)	−1.43* (−1.95 to −0.96)
						2015–2023	1.57 (−0.40 to 7.27)	
West	5.94		4.46	5.10		1999–2018	−2.13* (−2.76 to −1.43)	−1.35* (−1.85 to −0.86)
						2018–2021	7.82 (−4.03 to 10.48)	
						2021–2023	−6.86 (−12.67 to 2.48)	
Ten-year age cohorts								
45–54 yr	2.82		2.62	2.58		1999–2018	−1.30* (−1.83 to −0.85)	−0.32 (−0.83 to 0.13)
						2018–2021	12.46* (5.65–15.83)	
						2021–2023	−8.59 (−14.81 to 0.14)	
55–64 yr	4.31		3.98	3.83		1999–2007	−3.18* (−6.53 to −2.01)	−0.51* (−0.92 to −0.17)
						2007–2018	0.07 (−0.88 to 1.29)	
						2018–2021	9.43* (5.78–11.72)	
						2021–2023	−6.86* (−11.29 to −1.63)	
65–74 yr	7.22		5.13	5.71		1999–2001	3.61 (−1.29 to 8.72)	−1.25* (−1.57 to −0.89)
						2001–2008	−4.77* (−8.00 to −3.87)	
						2008–2018	−1.63* (−2.46 to −0.52)	
						2018–2021	7.90* (4.90–9.84)	
						2021–2023	−4.59* (−8.15 to −0.63)	
75–84 yr	12.43		7.74	9.96		1999–2002	2.39 (−0.83 to 7.63)	−1.85* (−2.15 to −1.54)
						2002–2016	−3.80* (−4.57 to −3.40)	
						2016–2023	0.34 (−0.89 to 2.08)	
85+ yr	26.62		15.03	20.16		1999–2002	0.90 (−3.30 to 7.32)	−2.19* (−2.70 to −1.70)
						2002–2018	−3.79* (−7.47 to −3.33)	
						2018–2023	1.23 (−1.84 to 7.38)	
Sex								
Females	5.08		3.65	4.13		1999–2016	−2.85* (−3.99 to −2.25)	−1.37* (−1.88 to −0.91)
						2016–2023	2.33 (−0.04 to 9.81)	
Males	7.84		5.71	6.45		1999–2002	1.19 (−0.71 to 4.37)	−1.25* (−1.46 to −1.02)
						2002–2007	−4.43* (−6.45 to −3.33)	
						2007–2018	−1.65* (−2.08 to −1.11)	
						2018–2021	7.24* (5.34–8.53)	
						2021–2023	−6.59* (−9.06 to −4.29)	
Alcoholic vs nonalcoholic								
Alcoholic	0.18		0.34		0.27	1999–2005	2.46 (−19.36 to 12.22)	3.75* (2.39–5.35)
						2005–2008	36.89 (−15.71 to 51.85)	
						2008–2011	−25.43 (−30.64 to 7.89)	
						2011–2023	5.79* (2.40–9.69)	
Nonalcoholic	0.92		4.37		3.50	1999–2003	−11.12 (−50.12 to 14.48)	6.16* (4.31–9.51)
						2003–2006	93.76* (49.23–142.54)	
						2006–2023	−0.46 (−1.79 to 0.76)	
Acute vs chronic pancreatitis								
Acute pancreatitis	5.33	3.7			4.34	1999–2001	3.90 (−0.15 to 7.81)	−1.42* (−1.69 to −1.14)
						2001–2007	−3.97* (−6.09 to −3.15)	
						2007–2018	−2.40* (−2.90 to −1.12)	
						2018–2021	7.00* (4.26–8.63)	
						2021–2023	−5.46* (−8.80 to −1.90)	
Chronic pancreatitis	1.05	1.05			0.96	1999–2004	0.68 (−5.46 to 9.43)	0.36 (−0.27 to 1.06)
						2004–2007	−8.55 (−12.13 to 5.06)	
						2007–2014	0.36 (−9.20 to 8.03)	
						2014–2023	3.34 (−3.70 to 9.30)	

Joinpoint regression identified distinct periods of decline, a transient reversal during 2018 to 2021, and heterogeneous patterns across subgroups, summarized using annual percent change (APC) and average annual percent change (AAPC) with 95% confidence intervals.

AAMR = age-adjusted mortality rate, CI = confidence interval.

* Demonstrates statistical significance.

Joinpoint analysis identified an initial increase from 1999 to 2001, followed by a sustained decline through 2018. A transient increase was observed between 2018 and 2021, after which mortality declined again through 2023.

### 3.2. Sex-specific mortality trends

Among females, the AAMR declined from 5.08 in 1999 to 3.65 in 2023, with a mean AAMR of 4.13. Mortality declined steadily from 1999 to 2016 (APC: −2.85%, 95% CI: −3.99 to −2.25), followed by a nonsignificant increase from 2016 to 2023 (APC: 2.33%, 95% CI: −0.04 to 9.81). Across the full study period, female mortality declined overall (AAPC: −1.37%, 95% CI: −1.88 to −0.91). The AAMR in males declined from 7.84 in 1999 to 5.71 in 2023, with a mean AAMR of 6.45. Mortality increased modestly from 1999 to 2002, declined significantly from 2002 to 2018, increased between 2018 and 2021 (APC: 7.24%, 95% CI: 5.34 to 8.53), and declined again from 2021 to 2023 (APC: −6.59%, 95% CI: −9.06 to −4.29). Over the full period, male mortality declined significantly (AAPC: −1.25%, 95% CI: −1.46 to −1.02).

Throughout the study period, pancreatitis-associated mortality remained consistently higher among males than among females. Both sexes exhibited long-term declines, with a shared increase during 2018 to 2021, followed by declines through 2023 (Fig. 1).

### 3.3. Mortality by age group

A pronounced age gradient was observed. The lowest mortality burden occurred among adults aged 45 to 54 years (AAMR: 2.58), while the highest burden was observed among those aged ≥85 years (AAMR: 20.16). Intermediate mortality was observed among individuals aged 75 to 84 years (AAMR: 9.96), 65 to 74 years (AAMR: 5.71), and 55 to 64 years (AAMR: 3.83).

Significant long-term declines were observed in all age groups except those aged 45 to 54 years. The steepest decline occurred among individuals aged ≥85 years (AAPC: −2.19%, 95% CI: −2.70 to −1.70), followed by those aged 75 to 84 years (AAPC: −1.85%, 95% CI: −2.15 to −1.54) and 65 to 74 years (AAPC: −1.25%, 95% CI: −1.57 to −0.89). Mortality among adults aged 55 to 64 years declined modestly (AAPC: −0.51%, 95% CI: −0.92 to −0.17), while no significant change was observed among those aged 45 to 54 years (AAPC: −0.32%, 95% CI: −0.83 to 0.13).

### 3.4. Mortality by race and ethnicity

#### 3.4.1. Trends from 1999 to 2017

During this period, the highest mortality burden was observed among Black or African American individuals (AAMR: 7.69), followed by American Indian or Alaska Native individuals (AAMR: 6.84) and White individuals (AAMR: 5.24). Lower mortality was observed among Hispanic or Latino individuals (AAMR: 4.65) and Asian or Pacific Islander individuals (AAMR: 3.40). Significant long-term declines were observed among most groups, with the steepest declines among Asian or Pacific Islander individuals (AAPC: −5.04%), Black or African American individuals (AAPC: −4.16%), and Hispanic or Latino individuals (AAPC: −3.63%). Declines among White individuals were more modest (AAPC: −1.36%). No significant change was observed among American Indian or Alaska Native individuals.

#### 3.4.2. Trends from 2018 to 2023

From 2018 to 2023, the highest mortality burden was observed among American Indian or Alaska Native individuals (AAMR: 7.33), followed by Black or African American individuals (AAMR: 5.81) and White individuals (AAMR: 5.04). Lower mortality was observed among Asian individuals (AAMR: 2.13) and individuals of more than 1 race (AAMR: 2.51). During this period, a significant decline was observed among Hispanic or Latino individuals (AAPC: −12.74%), while no statistically significant changes were observed among other racial and ethnic groups.

### 3.5. Mortality by US Census region

The highest overall mortality burden occurred in the South (AAMR: 5.72), followed by the Midwest (AAMR: 5.36) and the West (AAMR: 5.10). The Northeast exhibited the lowest burden (AAMR: 4.23). All regions experienced significant long-term declines, with the steepest decrease in the Northeast (AAPC: −1.75%) and the slowest decline in the Midwest (AAPC: −1.09%; Fig. 1).

### 3.6. Place of death

Most pancreatitis-associated deaths occurred in healthcare settings. Inpatient medical facilities accounted for 103,341 deaths (66.1%), while outpatient or emergency department settings accounted for 5687 deaths (3.6%). An additional 463 deaths (0.3%) were recorded as dead on arrival. Outside healthcare settings, 25,219 deaths (16.1%) occurred at home, 11,444 deaths (7.3%) occurred in nursing homes or long-term care facilities, and 5764 deaths (3.7%) occurred in hospice facilities.

### 3.7. Alcoholic and nonalcoholic pancreatitis

#### 3.7.1. Alcoholic pancreatitis

Between 1999 and 2023, 8504 deaths were attributed to alcoholic pancreatitis. The AAMR increased from 0.18 in 1999 to 0.34 in 2023. Mortality increased significantly from 2011 through 2023 (APC: 5.79%, 95% CI: 2.40–9.69), with a significant overall increase across the full study period (AAPC: 3.75%, 95% CI: 2.39–5.35; Fig. 2).

#### 3.7.2. Nonalcoholic pancreatitis

A total of 111,822 deaths were attributed to nonalcoholic pancreatitis. Mortality declined in the early study period, followed by a sharp increase between 2003 and 2006, after which rates remained relatively stable through 2023. Across the full study period, nonalcoholic pancreatitis mortality increased overall (AAPC: 6.16%, 95% CI: 4.31–9.51; Fig. 2).

In age-stratified analyses, nonalcoholic pancreatitis mortality increased consistently across all evaluable age groups, with rising crude rates observed within each stratum. These increases were accompanied by a substantially higher number of deaths compared with alcoholic pancreatitis, and by parallel increases in absolute death counts over time. The concordance between age-specific rate increases and rising mortality counts suggests that the observed increase in nonalcoholic pancreatitis mortality is not solely attributable to age standardization but reflects a broader increase across age groups (Table 2).

**Table 2:** Age-stratified crude mortality rates (CR) and average annual percent change (AAPC) with 95% confidence intervals (CI) for alcoholic and nonalcoholic pancreatitis among US adults aged ≥45 years (1999–2023), demonstrating consistent increases across age groups and a substantially higher mortality burden in nonalcoholic pancreatitis, particularly among older adults.

Age cohort	Deaths	CR in 1999	CR in 2023	Average CR	AAPC	Lower CI (AAPC)	Upper CI (AAPC)
Alcoholic pancreatitis							
45–54 yr	3433	0.17	0.4	0.32	5.52*	4.15	7.66
55–64 yr	3131	0.18	0.43	0.33	5.78*	4.44	9.37
65–74 yr	1367	0.16	0.3	0.22	3.44*	2.15	5.22
Nonalcoholic pancreatitis							
45–54 yr	18,305	0.64	2.26	1.72	5.36*	3.87	7.73
55–64 yr	25,221	0.87	3.58	2.63	5.79*	4.11	8.67
65–74 yr	25,086	0.95	4.85	3.83	6.70*	4.74	10.30
75–84 yr	23,709	1.24	7.59	6.63	6.89*	4.48	11.76
85+ yr	19,501	2.62	14.96	13.43	6.69*	4.06	11.89

* Indicates statistical significance.

### 3.8. Acute and chronic pancreatitis

#### 3.8.1. Acute pancreatitis

Between 1999 and 2023, 129,130 deaths were attributed to acute pancreatitis. The AAMR declined from 5.33 in 1999 to 3.70 in 2023, with a significant overall decline (AAPC: −1.42%, 95% CI: −1.69 to −1.14). A transient increase was observed between 2018 and 2021, followed by a decline through 2023 (Fig. 3).

#### 3.8.2. Chronic pancreatitis

Chronic pancreatitis accounted for 29,585 deaths from 1999 to 2023. The AAMR remained stable over the study period (1.05 in both 1999 and 2023), with no significant overall change (AAPC: 0.36%, 95% CI: −0.27 to 1.06; Fig. 3).

## 4. Discussion

In this national analysis of US death certificate data from 1999 to 2023, pancreatitis-associated mortality among adults aged ≥45 years declined overall, with the AAMR decreasing by more than 25%. This long-term improvement aligns with broader advances in pancreatitis care over the past 2 decades, including earlier recognition of severe disease, more standardized resuscitation and supportive strategies, improved critical care practices, and better prevention and management of complications.^[10]^ Against this sustained decline, mortality rose between 2018 and 2021 before falling again through 2023. This pattern is consistent with a temporary disruption of progress rather than a sustained reversal and may reflect a combination of system-level healthcare strain, delayed presentation, and shifting population risk profiles during the late 2010s and early pandemic period.^[11–13]^

Sex-stratified analyses showed persistently higher pancreatitis-associated mortality among males compared with females across the study period, despite substantial long-term declines in both groups. This gap is consistent with established epidemiologic patterns and may relate to differences in exposure to major risk factors, including alcohol use, tobacco exposure, and recurrent disease, as well as differences in severity at presentation and comorbidity burden.^[14,15]^ Notably, males also experienced a slightly greater overall decline in age-adjusted mortality across the full study period. This steeper reduction may reflect greater absolute benefit from improvements in pancreatitis management among higher-risk populations, including enhanced critical care practices, earlier recognition of severe disease, and targeted reductions in alcohol-related morbidity over time.^[16–18]^ Males also demonstrated greater short-term volatility in the late period, with a marked rise from 2018 to 2021 followed by a sharp decline afterward, whereas female mortality declined through 2016 and then plateaued. These differing trend structures suggest that males may be more sensitive to short-term shifts in risk exposure and healthcare access, superimposed on an otherwise improving long-term trajectory.^[19,20]^

A strong age gradient was evident, with substantially higher mortality in older age groups and the highest burden among adults aged ≥85 years. This finding is clinically intuitive, as older adults have lower physiologic reserve and are more vulnerable to complications such as organ failure, infection, and sepsis when pancreatitis occurs.^[21]^ At the same time, the steepest long-term declines occurred in the oldest groups, suggesting that improvements in inpatient and critical care may have yielded the largest absolute gains among those at the highest baseline risk. In contrast, younger and middle-aged adults exhibited lower baseline mortality and more modest or nonsignificant declines, consistent with a ceiling effect whereby already low fatality rates limit the magnitude of observable improvement over time.^[22]^ The absence of a significant long-term decline among adults aged 45 to 54 years may additionally reflect a higher proportion of alcohol-related pancreatitis and recurrent disease in this group, which can attenuate gains from advances in acute care.^[23,24]^

Pancreatitis-associated mortality varied substantially across racial and ethnic groups both before and after 2018, with persistently higher burdens observed among Black or African American and American Indian or Alaska Native individuals, and consistently lower mortality among Hispanic or Latino and Asian or Pacific Islander populations. These patterns likely reflect differences in underlying disease etiology and exposure, particularly a higher prevalence of alcohol-related and recurrent pancreatitis in groups with elevated mortality, compounded by greater comorbidity burden and reduced access to timely acute and preventive care.^[1,25]^ In contrast, lower baseline risk and fewer alcohol-related presentations among Hispanic or Latino and Asian or Pacific Islander individuals may have allowed broader advances in supportive and critical care to translate more effectively into long-term survival gains.^[25,26]^ During the pandemic era, these differences persisted, with American Indian or Alaska Native communities continuing to experience the highest mortality, while most other groups showed little evidence of further improvement, suggesting that healthcare disruptions may have stalled progress.^[27,28]^ The marked decline observed among Hispanic or Latino individuals after 2021 is notable but should be interpreted cautiously given limited data availability. Overall, these findings indicate that recent gains in pancreatitis outcomes have been uneven across populations, shaped by differences in risk exposure, comorbidity, and access to care, and emphasize the need for targeted strategies that address both acute management and upstream determinants of disease burden.^[1]^

Marked regional variation in pancreatitis-associated mortality was observed across the United States, despite consistent long-term declines in all census regions. The highest mortality burden in the South, followed by the Midwest, contrasts with the persistently lower rates in the Northeast and points to underlying regional differences in risk exposure and health system capacity. The elevated burden in the South and Midwest likely reflects a higher prevalence of alcohol use disorders, metabolic comorbidities, and chronic disease burden, as well as greater rural residency and barriers to timely access to specialized acute and critical care.^[29–31]^ In contrast, the Northeast’s consistently lower mortality and steepest rate of decline may be attributable to higher population density, shorter distances to tertiary care centers, greater availability of gastroenterology and critical care services, and earlier adoption of standardized pancreatitis management pathways.^[32,33]^ The slower pace of improvement observed in the Midwest suggests that structural and geographic factors may have limited the full translation of advances in pancreatitis care into mortality reductions, despite overall national progress.^[34]^

Place-of-death analysis indicated that pancreatitis-related mortality was predominantly hospital-based, consistent with the clinical course of fatal pancreatitis, which is characterized by rapid deterioration, persistent organ failure, and infectious complications requiring advanced inpatient and intensive care support.^[35]^ A smaller proportion of deaths occurred in outpatient, emergency department, or dead-on-arrival settings, likely reflecting abrupt clinical decline or delayed presentation.^[2]^ Notably, a meaningful share of deaths occurred at home and in nursing homes or long-term care facilities, suggesting that pancreatitis-associated mortality also arises in the setting of advanced comorbidity, frailty, or recurrent disease, particularly among individuals with chronic pancreatitis.^[1]^ Deaths in hospice settings likely reflect end-of-life care in patients with severe chronic pancreatitis or substantial competing illnesses.^[1]^

The contrasting mortality patterns observed for alcoholic and nonalcoholic pancreatitis suggest that these entities are influenced by different underlying drivers. Alcoholic pancreatitis showed a sustained increase in mortality beginning in the early 2010s, which may reflect cumulative alcohol exposure, recurrent disease episodes, and progression to chronic pancreatitis with associated complications.^[36–38]^ Individuals with alcohol-related disease often have delayed presentation, reduced engagement with preventive care, and a higher burden of comorbid conditions, all of which may contribute to poorer outcomes over time.^[39]^ In contrast, nonalcoholic pancreatitis accounted for the majority of deaths and demonstrated a sharp increase in the early 2000s, followed by relative stabilization thereafter. This pattern may be related to changes in diagnostic practices, improved recognition of pancreatitis as a contributing cause of death, and the growing prevalence of medical and procedural risk factors such as gallstone disease, metabolic conditions, medication exposure, and post-procedural complications.^[1,40]^ The absence of continued growth in nonalcoholic mortality after the mid-2000s suggests that the initial rise was driven more by attribution and detection rather than a persistent increase in disease severity.^[41,42]^ Although nonalcoholic pancreatitis represents a residual category, the consistency of increases across age strata and the parallel rise in crude rates and death counts suggest that the observed trend is unlikely to be explained solely by age-adjustment artifacts. Instead, these findings support a broader epidemiologic increase, particularly among older adults with higher baseline mortality risk.

Acute pancreatitis mortality declined steadily over most of the study period, with a significant overall reduction from 1999 to 2023, consistent with sustained improvements in clinical management and supportive care. The prolonged decline from the early 2000s through 2018 likely reflects earlier diagnosis, broader use of cross-sectional imaging, standardized severity assessment, improved critical care, and advances in infection control and organ support.^[43,44]^ The temporary increase observed between 2018 and 2021 likely represents an interruption of this trend and may be related to higher disease severity at presentation, delayed access to care, or broader system-level disruptions during this period.^[45]^ The subsequent decline after 2021 suggests partial recovery of care delivery and management pathways. In contrast, mortality attributable to chronic pancreatitis remained largely stable over the 25-year study period, with no significant overall change despite modest short-term fluctuations. This stability likely reflects the chronic, slowly progressive nature of the disease, in which mortality is more often driven by long-term complications than by acute deterioration.^[46]^ Advances in pain control, nutritional support, endoscopic interventions, and management of pancreatic insufficiency may have offset increasing exposure to risk factors such as alcohol use and metabolic disease, resulting in a relatively flat mortality trend.^[47,48]^ The absence of a sustained increase further suggests that improvements in outpatient management and complication surveillance may have limited fatal outcomes, even as the burden of chronic pancreatitis persists.^[49]^

### 4.1. Public health implications

The sustained decline in pancreatitis-associated mortality over the past 2 decades suggests that advances in acute management, critical care, and complication prevention have produced meaningful population-level gains. The temporary increase observed between 2018 and 2021, however, highlights the fragility of these improvements and the sensitivity of pancreatitis outcomes to disruptions in timely access to hospital-based and intensive care. Persistent disparities by sex, age, race and ethnicity, and region indicate that these benefits have not been evenly distributed, likely reflecting differences in risk exposure, comorbidity burden, and access to high-quality care. The continued rise in alcoholic pancreatitis mortality further underscores the limits of acute care alone and points to the need for stronger alcohol-focused prevention strategies, including population-level policy measures and improved access to addiction treatment. In contrast, the stabilization of nonalcoholic pancreatitis mortality suggests that gains in recognition and management may have tempered earlier increases, although the absolute burden remains substantial. Finally, the occurrence of a meaningful proportion of deaths outside acute care settings highlights the contribution of chronic disease, frailty, and multimorbidity to pancreatitis-related mortality and supports more integrated care models that link inpatient treatment with outpatient follow-up, long-term complication management, and palliative care when appropriate.

### 4.2. Strengths and limitations

This study has several notable strengths. Foremost, it leverages a large, nationally representative dataset spanning 25 years, allowing for robust assessment of long-term mortality trends and subgroup disparities. The use of multiple cause-of-death data provides a more comprehensive evaluation of pancreatitis-associated mortality than analyses restricted to the underlying cause of death, capturing cases in which pancreatitis contributed meaningfully to fatal outcomes but was not designated as the primary cause.^[50,51]^ Additionally, the application of age-adjusted rates and Joinpoint regression enables nuanced characterization of temporal patterns, including identification of inflection points and short-term deviations from long-standing trends. The extension of analyses through 2023 offers timely insight into recent mortality patterns, including the period encompassing major healthcare system disruptions.

Several limitations should also be considered. First, this analysis relies on death certificate data, which are subject to misclassification and variability in clinician reporting and coding practices. Inaccuracies on death certificates are common and can affect national mortality statistics, including errors in cause-of-death coding that alter the underlying cause recorded and influence mortality trends.^[52]^ Second, CDC WONDER does not include individual-level clinical information, such as disease severity, detailed etiology, treatment history, comorbidities, or socioeconomic status, limiting causal inference and preventing adjustment for important confounders in trend and disparity analyses.^[53]^ Third, the categorization of alcoholic versus nonalcoholic pancreatitis based on ICD-10 coding is inherently imperfect, as alcohol-related disease may be underreported or incompletely captured on death certificates. Nonalcoholic pancreatitis was defined as a residual category based on the absence of alcohol-related coding and likely includes heterogeneous etiologies. Although additional analyses support a true increase in mortality, we cannot fully exclude the influence of coding variability or misclassification in multiple cause-of-death data.^[54]^ Fourth, analyses were restricted to adults aged ≥45 years to improve estimate stability and reduce data suppression, which limits generalizability to younger populations in whom risk exposures and etiologic patterns may differ from older adults.^[4]^

Despite these limitations, the consistency of findings across multiple demographic and geographic strata, the use of standardized national data, and the alignment of observed trends with known clinical and epidemiologic patterns support the validity of the conclusions. Collectively, this study provides an updated and comprehensive assessment of pancreatitis-associated mortality in the United States, highlighting both sustained progress and persistent gaps that warrant focused public health and clinical attention.

## 5. Conclusion

Pancreatitis-associated mortality in the United States declined from 1999 to 2023, but this long-term improvement was interrupted by a temporary reversal between 2018 and 2021. Persistent disparities across sex, age, race and ethnicity, and region, together with rising alcohol-related pancreatitis mortality and largely stable chronic pancreatitis mortality, highlight the need for targeted prevention efforts, equitable access to care, and sustained advances beyond acute management.

## Author contributions

**Conceptualization:** Rateeba Qadri, Muzamil Khan.

**Project administration:** Rateeba Qadri, Muzamil Khan.

**Formal analysis:** Hasiba Karimi.

**Validation:** Hasiba Karimi, Usman Itoo, Dhruvan Patel.

**Methodology:** Usman Itoo.

**Investigation:** Hasibullah Aminpoor.

**Supervision:** Dhruvan Patel.

**Writing – original draft:** Rateeba Qadri, Muzamil Khan, Hasiba Karimi, Usman Itoo, Hasibullah Aminpoor.

**Writing – review & editing:** Rateeba Qadri, Muzamil Khan, Hasiba Karimi, Usman Itoo, Hasibullah Aminpoor.
